# Complete genome sequence of acute viral necrosis virus associated with massive mortality outbreaks in the Chinese scallop, *Chlamys farreri*

**DOI:** 10.1186/1743-422X-10-110

**Published:** 2013-04-08

**Authors:** Weicheng Ren, Haixia Chen, Tristan Renault, Yuyong Cai, Changming Bai, Chongming Wang, Jie Huang

**Affiliations:** 1 Maricultural Organism Disease Control and Pathogenic Molecular Biology Laboratory, Yellow Sea Fisheries Research Institute, Chinese Academy of Fishery Science, Qingdao, 266071, China; 2 Department of Rheumatology and Inflammation, University of Gothenburg, Gothenburg, 40530, Sweden; 3 Department of Biological and Environmental Sciences, University of Gothenburg, Gothenburg, 40530, Sweden; 4 Ifremer, Unité Santé, Génétique et Microgiologie des Mollusques, Laboratoire de Génétique et Pathologie des Mollusques Marins, La Tremblade, 17390, France

**Keywords:** Acute viral necrosis virus (AVNV), Herpesvirus, OsHV-1, Genome

## Abstract

**Background:**

Acute viral necrosis virus (AVNV) is the causative agent of a serious disease resulting in high mortality in cultured Chinese scallops, *Chlamys farreri*. We have sequenced and analyzed the complete genome of AVNV.

**Results:**

The AVNV genome is a linear, double-stranded DNA molecule of 210,993 bp with a nucleotide composition of 38.5% G + C. A total of 123 open reading frames were predicted to encode functional proteins, ranging from 41 to 1,878 amino acid residues. The DNA sequence of AVNV is 97% identical to that of ostreid herpesvirus 1 (OsHV-1), and the amino acid sequences of the encoded proteins of these two viruses are 94-100% identical. The genomic organization of AVNV is similar to that of OsHV-1, and consists of two unique regions (170.4 kb and 3.4 kb, respectively), each flanked by two inverted repeats (7.6 kb and 10.2 kb, respectively), with a third unique region (1.5 kb) situated between the two internal repeats.

**Conclusions:**

Our results indicate that AVNV is a variant of OsHV-1. The AVNV genome sequence provides information useful for understanding the evolution and divergence of OsHV-1 in marine molluscs.

## Background

Although viral infection in marine molluscs is a relatively young science, pathogens of this type have been reported worldwide in association with massive mortality outbreaks in economically significant species. Massive mortality of Portuguese oysters, *Crassostrea angulata*, in French stocks from 1967 to 1973 was associated with irido-like virus infections [1,2]. Other viruses infecting molluscs were interpreted as being members of the families *Togaviridae*, *Retroviridae*, *Reoviridae*, *Birnaviridae* or *Picornaviridae*[3-9]. Disseminated neoplasia, which was a proliferative cell disorder of the circulatory system in bivalves, was linked to the retroviral infections [10]. However, mollusc virology is still in its infancy and is based largely on morphological features because relevant biological and molecular tools are scarce.

Herpesviruses comprise an abundant, widely distributed group of large DNA viruses in vertebrates and invertebrates, including mammals, birds, reptiles, fish and marine molluscs. They were classified into the families *Alloherpesviridae*, *Herpesviridae* and *Malacoherpesviridae* in the order *Herpesvirales*[11]. The genomes of herpesvirus have been accumulating since the 1980s, and sixty-eight isolated from different species have been deposited in GenBank to date. These genomes have been interpreted to give detailed views of ubiquitous and lineage-specific functions. Herpesviruses and herpes-like viruses have also been attracted particular attention because of their ecological and economic impact on wild and cultured marine molluscs during the last 20 years, and several were reported worldwide [12-27]. The term herpes-like virus tends to be used when a virus has been characterized exclusively on the basis of morphological features. One herpesvirus that infects Pacific oysters, *Crassostrea gigas*, in France, has been fully characterized on both morphological and molecular basis. This virus was named ostreid herpesvirus 1 and was classified as the founding member of the species *Ostreid herpesvirus 1*, genus *Ostreavirus*, family *Malacoherpesviridae*[11,25]. Although OsHV-1 was first described in the larvae of Pacific oysters in France, further studies have demonstrated that it was able to infect other bivalve species, including Manila clam, *Ruditapes philippinarum*[27], and French scallop, *Pecten maximus*[28]. Recently, a distinct OsHV-1 genotype (OsHV-1 μVar) has been also reported in association with massive mortality in Pacific oysters in France [29].

Since the mid-1990s, the farming of Chinese scallops has experienced a period of severe crisis due to the ongoing mortality outbreaks. The disease has occurred annually in summer and mortality reaches more than 90% within 5–8 days after first appearance [30,31]. The causative agent was determined to be a virus and was named acute viral necrosis virus (AVNV) [32,33]. According to the previous data, AVNV seems to be related to OsHV-1 based on morphology [33,34], histopathological features such as basophilic inclusions and cellular changes [34,35], and epidemiological aspects [36]. Therefore, based on the published OsHV-1 DNA sequences as a template, we have sequenced the complete genome of AVNV and have carried out the comparative analysis.

## Results

### Determination of the AVNV genome sequence

Because no reliable cell lines are available for the propagation and isolation of AVNV, a PCR-based approach was used to obtain the complete genomic DNA sequences. Initially, OsHV-1 specific primers A3/A4 [26], C2/C6 [28] and Gp3/Gp4 [28] were used to amplify AVNV DNA sequences, which were then compared to the corresponding OsHV-1 sequences. The results showed that these three AVNV fragments were 99%, 97% and 99% identical to OsHV-1, respectively (data not shown), thus indicating that the DNA sequences of these two viruses may be generally highly similar. We then extended the PCR-based method to sequence the whole genome of AVNV by designing primers based on the OsHV-1 DNA sequences.

**Figure 1:**
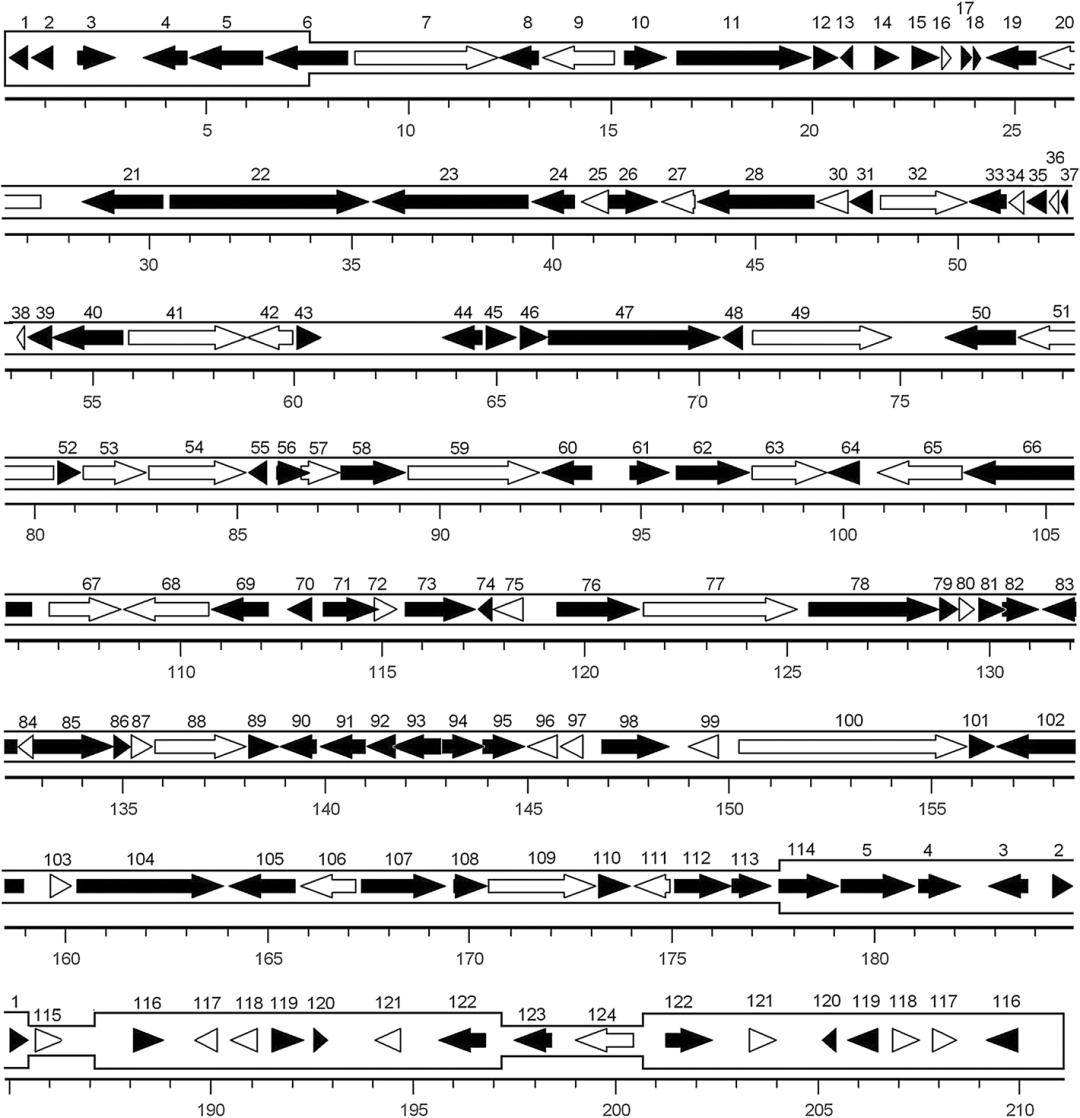
**Organization of the AVNV genome.** Arrows indicate the location and orientation of the ORFs. White arrows represent ORFs with predicted functions similar to those in other herpesviruses, and black arrows represent ORFs with unknown functions. The two inverted repeats (ORF1-ORF6 and ORF116-ORF122) are shown in a thicker format. The scale is in kb.

The AVNV genome was initially determined to be 210,825 bp in size. However, a large palindrome located correspondingly between ORF49 and ORF50 in the OsHV-1 genome appeared to be deleted upon cloning into the plasmids used for sequencing [25]. Palindromes that are deleted in similar circumstances were also reported in members of the subfamily *Alphaherpesvirinae*[37,38] and in the genus *Roseolovirus* of the subfamily *Betaherpesvirinae*[39]. We resolved the sequence of this region in AVNV by using the method described by Weller *et al.*[38]. Finally, the complete AVNV genomic DNA sequences was determined to be 210,993 bp, and had a nucleotide composition of 38.5% G+C. The structure of the AVNV genome consisted of two unique regions (170.4 kb and 3.4 kb, respectively), each flanked by an inverted repeat (7.6 and 10.2 kb, respectively), with the internal copies of the repeats separated by a third unique 1.5 kb region (Figure 1).

### Coding capacity of the AVNV genome

Analysis of the AVNV genome resulted in the prediction of 123 unique open reading frames (ORFs) potentially encoding functional proteins and ranging in size from 41 to 1,878 amino acid residues (Additional file 1: Table S1). Owing to the presence of the inverted repeats, 12 ORFs were duplicated, resulting in a total of 135 putative genes in AVNV. The ORFs on the upper (R) and lower (L) DNA strands (53% lower, 47% upper) were numbered following the OsHV-1 nomenclature (Additional file 1: Table S1) and a diagrammatic representation of their arrangement was shown in Figure 1. The proportion of the genome encoding ORFs was about 82%, which was similar to that of OsHV-1 (84%). The average length of AVNV ORFs was 1,260 bp, which was marginally smaller than that of OsHV-1 (1,272 bp). Five pairs of overlapping ORFs were found in the AVNV genome, including ORF56 and ORF57, ORF71 and ORF72, ORF81 and ORF82, ORF92 and ORF93, and ORF94 and ORF95 (Figure 1), with the overlapping regions being 8–251 bp in size. ORF28 and ORF29 overlapped by 125 bp in OsHV-1 [25] but are present as a single ORF 28 in AVNV (Additional file 1: Table S1).

### Overall comparisons between the AVNV and OsHV-1 genomes

OsHV-1 was classified as the founding member of the family *Malacoherpesviridae*[11], which differs significantly from other herpesvirus families [40]. Therefore, we compared the genome sequence of AVNV with that of OsHV-1. The results showed that AVNV was similar to OsHV-1 in genome organization, DNA sequences and ORF layout (Figure 1). The majority of AVNV ORFs were closely matched in size and orientation with their OsHV-1 counterparts, with identities from 94% to 100% (Additional file 1: Table S1). The AVNV and OsHV-1 DNA sequences were also very similar, exhibiting about 97% identity overall. However, there were several obvious insertions and deletions between the two genomes, the most notable being located in the AVNV genome at positions 1,500-1,700, 60,700-63,350, 183,900-184,100, 187,300-190,300, 192,800-195,100, 203,000-205,100 and 207,800-210,700. All of these above variations were located in the non-coding regions.

### Comparisons of C2/C6 and Gp regions between AVNV and OsHV-1

**Figure 2:**
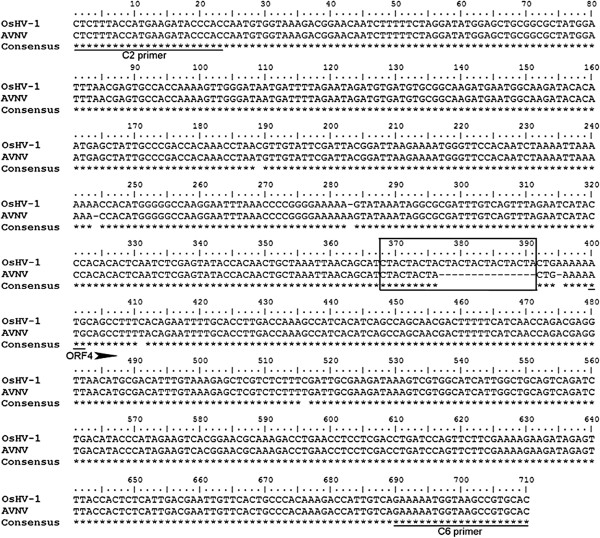
**DNA sequences alignment of the C2/C6 fragment in OsHV-1 and AVNV.** The location of primers C2 and C6 is underlined. The initiation codon (ATG) for ORF4 is underlined and marked by an arrowhead, and the region of CTA microsatellite is marked in the box.

To compare the DNA sequences of the two viruses further, two fragments that were frequently used to detect OsHV-1 by PCR were analyzed and compared. The C2/C6 fragment contains polymorphisms that have been used to differentiate several OsHV-1 genotypes [27-29]. In this region, the DNA sequence of AVNV was 97% identical to that of OsHV-1, differing by three deletions, one insertion, and two substitutions (Figure 2). The major deletion consisted of five copies of a trinucleotide repeat (CTA) that was described previously as being a microsatellite region [29]. This trinucleotide was repeated three times in AVNV and eight times in OsHV-1 (Figure 2). Compared to OsHV-1, there were also two deletions of A residues in AVNV at positions 244 and 395, an insertion of an A residue at position 283, and two synonymous substitutions in ORF4 at positions 411 and 516 (both C to T changes).

The Gp region encodes a putative glycoprotein (ORF88) and has been utilized to design primers for the detection and identification of OsHV-1 variants in French scallops [28]. The AVNV and OsHV-1 DNA sequences differed in this region by one synonymous and five non-synonymous substitutions (Table 1), yielding 99% identity in this region at both the nucleotide and amino acid sequence levels. The five non-synonymous substitutions induced the modifications of ACC (T, threonine), GTG (V, valine), GCG (A, alanine), ATC (I, isoleucine) and GAA (E, glutamate) codons to AGC (S, serine), GCG (A, alanine), ACG (T, threonine), CTC (L, leucine) and GAC (D, aspartate) codons, respectively (Table 1).

**Table 1:** Sequence variations in the Gp region (ORF88) between OsHV-1 and AVNV

**Position (bp)**	**Nucleotide substitution (OsHV-1 to AVNV)**	**Amino acid substitution (OsHV-1 to AVNV)**
254	C→G	T→S
1094	T→C	V→A
1821	A→G	No
1963	G→A	A→T
1969	A→C	I→L
1995	A→C	E→D

## Discussion

The complete genomic DNA sequences of AVNV isolated from Chinese scallops were determined using a PCR amplification strategy that has been used extensively to generate genome sequences for other viruses [41-43]. The genome of AVNV was 210,993 bp in size, which was slightly longer than that of OsHV-1, and had a nucleotide composition of 38.5% G+C, which is also similar to that of OsHV-1 (38.7%) The genome organization consisted of three unique regions and two inverted repeat regions, which was similar as that of OsHV-1 [25] and also similar as that of herpes simplex virus [44] and human cytomegalovirus [45]. Comparative analysis of sequences revealed that AVNV was highly related, but not identical, to OsHV-1 at the nucleotide and amino acid sequence levels (97% and 94-100%, respectively). In addition, previous reports showed that the two viruses were also similar in epidemiology [36,46,47], morphology [25,33,34,48] and histopathology [15,28,34,35]. Based on these results, we propose that AVNV may be a variant of OsHV-1.

Sequence comparisons have become the primary approach for evaluating phylogenetic and taxonomic relationships among herpesviruses and for identifying and assigning newly characterized viruses [40]. Using the C2/C6 PCR primers, several OsHV-1 variants were described in France in clams [27], Pacific oysters and scallops [27,28]. This fragment contains a polymorphic microsatellite region consisting of a number of CTA repeats. AVNV has three repeats, whereas OsHV-1 has eight [25], the variant OsHV-1 μVar has four [29], another OsHV-1 variant has six [49], and other French specimens present various numbers of CTA repeats [50]. These observations reinforce the fact that the microsatellite region does display polymorphisms and could be utilized for identifying and differentiating among OsHV-1 variants.

Generally, glycoproteins on the viral envelope bind to specific receptor molecules on the host cell, promoting viral entry into the host cell. For ORF88, encoding a putative glycoprotein [25,28], the modification of GTG (V, valine) to GCG (A, alanine) in AVNV was also reported in an OsHV-1 variant from French scallops by using the Gp3/Gp4 PCR primers to amplify a part of the ORF [28]. It is possible that the polymorphisms in this region might reflect the host-specific, and this needs further investigation. Nevertheless, the fact that OsHV-1 has more than one host species is different to the situation for most vertebrate herpesviruses, which are thought to have co-evolved or adapted in association with single host species, although exceptions have been described [40,51]. Upon successful transmission to new host species, viruses usually adapt quickly to the changed immunological environment [52]. One of the mechanisms of adaptation involves amino acid changes, in particular in proteins that may facilitate transmission [53]. Indeed, a number of proteins have been implicated in determining host specificity for various viruses [54]. For instance subtypes of influenza A virus may be distinguished by two surface glycoproteins, and amino acid substitutions may alter receptor binding to permit transmission from humans to birds [55,56].

In comparison to OsHV-1, AVNV presents a large number of variations including deletions, insertions and substitutions in both coding and non-coding regions. One region located at 60,700-63,350 bp in AVNV is particularly unusual in bearing a large insertion of 2.6 kb compared to the OsHV-1 genome. Several OsHV-1 genotypes have also been described in oysters, scallops and clams based on analysis of various genome regions [27-29,50,57]. The finding that OsHV-1 specimens collected from different locations may have similar DNA sequences [50,57], whereas others collected from the same place showed different genotypes [29], suggests that particular genotypes may be not distributed geographically. More work on genome sequences analysis of different OsHV-1 genotypes would be useful in defining additionally diagnostic polymorphisms. Moreover, sequencing more OsHV-1 strains from different locations and host species may help to elucidate the biological and pathogenic associations of the various genotypes.

## Conclusions

In this study, we have sequenced the AVNV genome sequence using a PCR-based approach. The AVNV genome is a linear, double-stranded DNA molecule of 210,993 bp and its organization and ORFs layout are similar to that of OsHV-1. The DNA and amino acid sequences of AVNV are 97% and 94-100% identical to that of OsHV-1, respectively. Therefore, together with previous observations, our results suggest that AVNV could be a variant of OsHV-1.

## Materials and methods

### Samples

AVNV infected scallops, *C. farreri*, were collected from Qingdao, China, in 2007. All diseased animals showed clinical signs including slow reactions, weak water-spray, shrunken mantle, blemished ocelli and an enlarged digestive gland. Virus particles were observed in specimens by electron microscopy.

### Purification of virus and viral DNA

Purification of virus particles was conducted as described by Wang *et al*. [33] and LeDeuff and Renault [48], with minor modifications. Seawater was filtered through 0.22 μm membranes (Millipore, USA) and used in the following purification steps. Mantle, gill and kidney tissues from scallops were rinsed 3 times and homogenized in seawater (1:9) using an Ultra-Turrax tissue homogenizer. After centrifugation at 3,500 × g and 7,500 × g for 15 min at 4°C, the supernatant was overlaid onto a 30% (w/v) sucrose solution and centrifuged at 125,000 × g for 1.5 h at 4°C. The pellet was resuspended in seawater by mixing gently. The virus suspension was then layered on a 30-55% (w/v) sucrose gradient and centrifuged at 125,000 × g for 3 h at 4°C. The viral band was removed from the tube by side puncture, and AVNV DNA was extracted using a Takara MiniBEST DNA Extraction Kit Ver. 3.0 (Takara Bio Dalian Co. Ltd. Dalian, China) according to the manufacturer’s protocol. The concentration of viral DNA was determined using a spectrophotometer.

### PCR amplification and DNA sequencing

The AVNV genome sequence was determined using a PCR-based approach. As the sequences of OsHV-1 were highly similar in AVNV, a total of 62 PCR primer pairs were designed based on the OsHV-1 genome sequence (GenBank accession AY509253) and used to amplify overlapping AVNV DNA fragments (from 600 to 5000 bp). The AVNV genome termini were identified using the method described by Davison et al. [25,45]. Briefly, AVNV DNA was treated with T4 DNA polymerase in the presence of the four dNTPs to produce flush ends, and ligated into the partially double-stranded adaptor (the cDNA adaptor in the Clontech Marathon kit). PCR was carried out using an adaptor-specific primer plus a primer specific for the left or right terminal region of the genome (5’-CACGGTGGGAAGGCTGAT-3’ or 5’-GATAGGAGGTTAGACACGC-3’ and Ex Taq polymerase (Takara). The products were purified using a TaKaRa gel purification kit (Takara), and inserted into pGEM-T (Promega, USA). The cloned fragments were sequenced in both directions using universal primers and an ABI PRISM 3770 (Applied Biosystems, Inc., USA). Additional primers (236 in total) were designed for sequencing the internal regions of longer PCR products (>1200 bp). At least three individual clones were sequenced for each fragment in order to exclude potential mutations generated by PCR.

### Computer-assisted analysis of DNA sequence data

Genomic composition and structure were analyzed using DNASTAR (Lasergene). The location and amino acid sequences of ORFs were predicted using Accelrys Gene 2.5 (Accelrys Inc.) and NCBI ORF finder (http://www.ncbi.nlm.nih.gov/gorf/gorf.html) according to the following criteria: (1) they were ≥120 bp in size, (2) they were not located within larger ORFs, (3) polyadenylation signal were analyzed and (4) they were compared with other sequences using NCBI BLASTP (http://www.ncbi.nlm.nih.gov/). Dot matrix comparisons of DNA sequences were carried out using Accelrys Gene 2.5. Complete genome sequence alignments were performed using Geneious (Biomatters Ltd, New Zealand).

### Nucleotide sequence accession number

The complete genome sequence of AVNV reported in this paper has been released in the GenBank database under accession number GQ153938.

## Abbreviations

AVNV: Acute viral necrosis virus; OsHV-1: Ostreid herpesvirus 1; ORF: Open reading frame; GP: Glycoprotein; bp: Base pair; kb: Kilobase pair; PCR: Polymerase chain reaction.

## Competing interests

The authors declare that they have no competing interests.

## Authors' contributions

WR and CW conceived the study and wrote the manuscript; YC participated in sample collection; TR, JH and HC participated in the discussion and modification of the manuscript; WR carried out the experiments and data analysis. HC and CB participated in the re-analysis of data when revised the manuscript. All authors have read and approved the manuscript.

## Supplementary Material

Additional file 1: Table S1 Potential open reading frames of the AVNV genome. Click here for file
