# Supplementary material for: Complete genome sequence of acute viral necrosis virus associated with massive mortality outbreaks in the Chinese scallop, Chlamys farreri
Source: Virol J. 2013 Apr 8;10:110. doi: 10.1186/1743-422X-10-110 (PMC3623871; doi:10.1186/1743-422X-10-110)
Supplement: Additional file 1: Table S1 — Potential open reading frames of the AVNV genome. [file 1743-422X-10-110-S1.docx]

Table 3

Potential open reading frames of the AVNV genome

| ORF | Position | Conserved domain or signature ^a^ (CD assession no.) | Predicted structure or function ^b^ | Identitles to OsHV-1 | |
| --- | --- | --- | --- | --- | --- |
|  |  |  |  | ORF | Identity |
| 001 | 114-560 |  |  | 001 | 99 |
| 002 | 677-1180 |  |  | 002 | 100 |
| 003 | 1816-2739 |  |  | 003 | 100 |
| 004 | 3453-4502 |  |  | 004 | 99 |
| 005 | 4598-6398 |  |  | 005 |  |
| 006 | 6475-8505 |  |  | 006 | 100 |
| 007 | 8681-12226 | Herpesviridae UL52/UL70 DNA primase (pfam03121) | Motifs V and VI characteristic of SF2 helicases | 007 | 99 |
| 008 | 12265-13224 |  |  | 008 | 99 |
| 009 | 13312-15090 | MoxR-like ATPases (COG0714) | RING finger protein | 009 | 100 |
| 010 | 15350-16376 |  |  | 010 | 99 |
| 011 | 16643-19930 |  |  | 011 | 99 |
| 012 | 20035-20622 |  |  | 012 | 98 |
| 013 | 20669-20986 |  |  | 013 | 100 |
| 014 | 21533-22117 |  |  | 014 | 98 |
| 015 | 22463-23107 |  |  | 015 | 99 |
| 016 | 23185-23400 |  | Membrane protein | 016 | 97 |
| 017 | 23672-23914 |  |  | 017 | 100 |
| 018 | 23966-24142 |  |  | 018 | 100 |
| 019 | 24294-25502 |  |  | 019 | 99 |
| 020 | 25582-27321 | Ribonucleotide reductase,  R2/beta subunit (RNRR2)  (cd01049) | Ribonucleotide reductase small subunit | 020 | 99 |
| 021 | 28332-30344 |  |  | 021 | 97 |
| 022 | 30510-35408 |  |  | 022 | 98 |
| 023 | 35540-39358 |  |  | 023 | 99 |
| 024 | 39455-40507 |  | Primase | 024 | 99 |
| 025 | 40671-41336 |  | Class I membrane protein | 025 | 100 |
| 026 | 41384-42571 |  |  | 026 | 99 |
| 027 | 42681-43481 | Dut, dUTPase (COG0756) | Inactive dUTPase | 027 | 99 |
| 028 | 43566-46421 |  |  | 028, 029 |  |
| 030 | 46524-47270 | Herpes_UL92, UL92 family (pfam03048) | Related via cysteine-rich domain to ORF31 of genus Rhadinovirus and UL92 of genus Cytomegalovirus of the Herpesviridae | 030 | 100 |
| 031 | 47324-47881 |  |  | 031 | 99 |
| 032 | 48141-50271 |  | Encodes class I membrane protein | 032 |  |
| 033 | 50283-51173 |  |  | 033 | 99 |
| 034 | 51253-51627 |  | Inactive dUTPase | 034 | 100 |
| 035 | 51713-52186 |  |  | 035 | 100 |
| 036 | 52256-52483 |  | Membrane protein | 036 | 100 |
| 037 | 52552-52677 |  |  | 037 | 100 |
| 038 | 52749-53286 | RING-finger-containing ubiquitin ligase (COG5540) | RING finger protein | 038 | 98 |
| 039 | 53426-54010 |  |  | 039 | 99 |
| 040 | 54013-55740 |  |  | 040 | 99 |
| 041 | 55890-58808 |  | Class I membrane protein | 041 | 99 |
| 042 | 58842-59936 | Baculoviral inhibition of apoptosis protein repeat domain (cd00022) | BIR protein containing RING finger | 042 | 99 |
| 043 | 60055-60666 |  |  | 043 | 100 |
| 044 | 63688-64614 |  |  | 044 | 99 |
| 045 | 64758-65483 |  |  | 045 | 100 |
| 046 | 65631-66278 |  |  | 046 | 99 |
| 047 | 66294-70532 |  |  | 047 | 99 |
| 048 | 70598-71080 |  |  | 048 | 99 |
| 049 | 71351-74767 | Herpesviridae UL52/UL70 DNA primase (pfam03121) | Contains motifs V and VI characteristic of SF2 helicases | 049 | 99 |
| 050 | 76128-77857 |  |  | 050 |  |
| 051 | 77941-80446 | RNR, class I. Ribonucleotide reductase (RNR) (cd01679) | Ribonucleotide reductase large subunit | 051 | 99 |
| 052 | 80563-81105 |  |  | 052 | 100 |
| 053 | 81189-82736 |  | RING finger-like protein | 053 | 99 |
| 054 | 82795-85218 |  | Class I membrane glycoprotein | 054 | 99 |
| 055 | 85295-85714 |  |  | 055 | 99 |
| 056 | 86021-86836 |  |  | 056 | 99 |
| 057 | 86586-87536 | Mid-1-related chloride channel (MCLC) (pfam05934) | Multiple transmembrane protein; chloride channel | 057 | 99 |
| 058 | 87581-89146 |  |  | 058 | 100 |
| 059 | 89239-92481 |  | Class I membrane protein | 059 | 99 |
| 060 | 92534-93769 |  |  | 060 | 100 |
| 061 | 94723-95682 |  |  | 061 | 97 |
| 062 | 95841-97641 |  |  | 062 |  |
| 063 | 97739-99585 |  | Encodes class I membrance protein | 063 |  |
| 064 | 99625-100377 | RNA_lig_T4_1 superfamily (pfam09511) | RNA ligase | 064 | 98 |
| 065 | 100819-102919 |  | Encodes class I membrance protein | 065 |  |
| 066 | 102974-106366 |  |  | 066 | 99 |
| 067 | 106779-108569 | DEAD-like helicases superfamily (cd00046). | SF2 helicase | 067 | 99 |
| 068 | 108649-110733 |  | Class I membrane protein | 068 | 98 |
| 069 | 110801-112192 |  |  | 069 | 100 |
| 070 | 112677-113279 |  |  | 070 | 99 |
| 071 | 113566-114927 |  |  | 071 | 99 |
| 072 | 114825-115391 |  | Membrane protein | 072 | 99 |
| 073 | 115609-117332 |  |  | 073 |  |
| 074 | 117385-117741 |  |  | 074 | 100 |
| 075 | 117766-118509 | dUTPase (pfam00692) | dUTPase | 075 | 95 |
| 076 | 119340-121376 |  |  | 076 | 99 |
| 077 | 121494-125285 |  | Class I membrane protein | 077 | 96 |
| 078 | 125570-128794 |  |  | 078 | 99 |
| 079 | 128808-129248 |  |  | 079 | 99 |
| 080 | 129312-129662 |  | Membrane protein | 080 | 100 |
| 081 | 129786-130427 |  |  | 081 | 100 |
| 082 | 130375-131265 |  |  | 082 | 100 |
| 083 | 131360-132466 |  |  | 083 | 99 |
| 084 | 132472-132828 |  | Membrane protein | 084 | 99 |
| 085 | 132833-134836 |  |  | 085 | 99 |
| 086 | 134840-135247 |  |  | 086 | 99 |
| 087 | 135262-135774 | Baculoviral inhibition of apoptosis protein repeat domain (cd00022) | BIR protein lacking RING finger | 087 | 100 |
| 088 | 135867-137113 |  | Class I membrane protein | 088 | 99 |
| 089 | 138166-138900 |  |  | 089 | 99 |
| 090 | 138961-139845 |  |  | 090 | 99 |
| 091 | 139962-141044 |  |  | 091 | 100 |
| 092 | 141094-141783 |  |  | 092 | 99 |
| 093 | 141719-142933 |  |  | 093 | 99 |
| 094 | 142938-143981 |  |  | 094 | 100 |
| 095 | 143974-144999 | Morph_protein1; Defects in morphology protein 1, mitochondrial precursor (pfam09810) | Similar to a family of uncharacterized, conserved eukaryotic proteins | 095 | 90 |
| 096 | 145064-145786 |  | RING finger protein | 096 | 100 |
| 097 | 145868-146413 |  | RING finger protein | 097 | 99 |
| 098 | 146916-148559 |  |  | 098 | 98 |
| 099 | 149043-149795 | Baculoviral inhibition of apoptosis protein repeat domain (cd00022) | BIR protein lacking RING finger | 099 | 99 |
| 100 | 150286-155922 | DNA polymerase family B; DNA-directed DNA polymerases (cd00145) | DNA polymerase | 100 | 99 |
| 101 | 156009-156638 |  |  | 101 | 99 |
| 102 | 156679-158967 |  |  | 102 | 96 |
| 103 | 159734-160252 |  | Multiple transmembrane protein | 103 | 99 |
| 104 | 160402-164004 |  |  | 104 | 99 |
| 105 | 164141-165755 |  |  | 105 |  |
| 106 | 165900-167294 | Baculoviral inhibition of apoptosis protein repeat domain (cd00022) | BIR protein containing RING finger | 106 | 97 |
| 107 | 167416-169485 |  |  | 107 | 99 |
| 108 | 169705-170517 |  |  | 108 | 99 |
| 109 | 170570-173194 | Probable DNA packing protein, C-terminus (pfam02499) | ATPase subunit of DNA-packaging terminase | 109 | 100 |
| 110 | 173289-174074 |  |  | 110 | 99 |
| 111 | 174164-175033 |  | Multiple transmembrane protein | 111 | 99 |
| 112 | 175157-176545 |  |  | 112 | 100 |
| 113 | 176552-177508 |  |  | 113 | 100 |
| 114 | 177713-179215 |  |  | 114 | 100 |
| 005 | 179291-181089 |  |  | 005 |  |
| 004 | 181188-182237 |  |  | 004 | 99 |
| 003 | 182951-183874 |  |  | 003 | 100 |
| 002 | 184510-185013 |  |  | 002 | 100 |
| 001 | 185130-185576 |  |  | 001 | 99 |
| 115 | 185763-186476 | Herpes_ori_bp, Origin of replication binding protein (pfam02399) | Contains motifs V and VI characteristic of SF2 helicases | 115 | 94 |
| 116 | 188199-188951 |  |  | 116 | 97 |
| 117 | 189695-190270 |  | RING finger protein | 117 | 98 |
| 118 | 190605-191273 | RING-finger (Really Interesting New Gene) domain (cd00162) | RING finger protein | 118 | 98 |
| 119 | 191642-192400 |  |  | 119 | 99 |
| 120 | 192662-192991 |  |  | 120 | 99 |
| 121 | 194152-194799 |  | RING finger protein | 121 | 97 |
| 122 | 195736-196890 |  |  | 122 | 99 |
| 123 | 197608-198519 |  |  | 123 | 100 |
| 124 | 199122-200546 |  | RING finger-like protein | 124 | 99 |
| 122 | 201303-202457 |  |  | 122 | 99 |
| 121 | 203394-204041 |  | RING finger protein | 121 | 97 |
| 120 | 205202-205531 |  |  | 120 | 99 |
| 119 | 205793-206551 |  |  | 119 | 99 |
| 118 | 206920-207588 |  | RING finger protein | 118 | 98 |
| 117 | 207923-208498 |  | RING finger protein | 117 | 98 |
| 116 | 209242-209994 |  |  | 116 | 97 |

^a^ Conserved domain or signature was constructed using the program CD-Search within BlastP.

^b^ Function was deduced from annotation of the OsHV-1 genome in the GenBank (AY509253).
